# ChunkUIE: Chunked instruction-based unified information extraction

**DOI:** 10.1371/journal.pone.0326764

**Published:** 2025-06-27

**Authors:** Wei Li, Yingzhen Liu, Yinling Yang, Ting Zhang, Wei Men

**Affiliations:** 1 National Defense University, Beijing, China; 2 State Key Laboratory of Geo-Information Engineering, Beijing, China; 3 Beijing Gengtu Technology Co., Ltd., Beijing, China; 4 School of Transportation, Southeast University, Nanjing, China; University of Passau: Universitat Passau, GERMANY

## Abstract

Large language models (LLMs) have demonstrated remarkable performance across various linguistic tasks. However, existing LLMs perform inadequately in information extraction tasks for both Chinese and English. Numerous studies attempt to enhance model performance by increasing the scale of training data. However, discrepancies in the number and type of schemas used during training and evaluation can harm model effectiveness. To tackle this challenge, we propose ChunkUIE, a unified information extraction model that supports Chinese and English. We design a chunked instruction construction strategy that randomly and reproducibly divides all schemas into chunks containing an identical number of schemas. This approach ensures that the union of schemas across all chunks encompasses all schemas. By limiting the number of schemas in each instruction, this strategy effectively addresses the performance degradation caused by inconsistencies in schema counts between training and evaluation. Additionally, we construct some challenging negative schemas using a predefined hard schema dictionary, which mitigates the model’s semantic confusion regarding similar schemas. Experimental results demonstrate that ChunkUIE enhances zero-shot performance in information extraction.

## 1 Introduction

In recent years, large language models (LLMs) have achieved significant breakthroughs across various tasks in natural language processing (NLP)[1–4]. Information extraction (IE) is one of the NLP tasks, aiming to extract explicit and structured knowledge from text. But some recent work indicates that LLMs’ performance has a significant gap in the IE [5–8]. In order to improve the performance of LLM on IE tasks. The Unified Information Extraction (UIE) [9] proposes a schema-based prompt mechanism that effectively facilitates the extraction of structured knowledge, including advanced entities, relationships, and events. Earlier models typically required the design of task-specific architectures for different information extraction tasks. However, UIE employs a single model that supports multiple tasks, such as named entity recognition (NER), relation extraction (RE), and event extraction (EE). Subsequent research has focused on schema design and dataset construction, demonstrating the effectiveness of employing schemas in information extraction tasks [10].

Current studies often simplify schema construction, which may adversely affect the performance of information extraction models. A common approach involves concatenating all schemas from the dataset to create a training set, allowing the inference process to utilize any subset of these schemas. This inconsistency between training and inference can undermine the model’s reasoning capabilities, while employing all schemas during inference is also impractical. To tackle this challenge, we propose a chunk-based schema instruction construction strategy.

Utilizing the state-of-the-art LLMs, Qwen2-7B-Chat [11] and Llama3.1-8B-Chat [12], we employ the LoRA [13] technique to conduct experiments on multiple Chinese and English datasets for NER, RE, and EE tasks to validate the performance improvements of our proposed method under zero-shot conditions. Experimental results show that discrepancies in schema distributions during training and inference can negatively impact model performance. Additionally, ablation studies indicate that focusing on challenging negative schemas enhances the model’s ability to distinguish similar patterns.

The main contributions of this study are summarized as follows:

1. We propose a chunk-based schema instruction construction method. This approach divides schemas using a carefully designed sampling strategy based on an explicitly specified chunk size, ensuring that the schema distributions during training and inference are as similar as possible. This alignment helps mitigate the adverse effects on model performance resulting from significant differences in schema patterns. Furthermore, our method constructs the training set in a comprehensive and explicit manner, enriching the variety of schema patterns included.
2. We introduce a predefined dictionary of challenging schemas. The schema segmentation process prioritizes the co-occurrence of schemas corresponding to positive label with those of challenging negative labels, thereby enhancing the model’s ability to discern similar semantics and reducing confusion over similar patterns.
3. Extensive experiments conducted on publicly availableChinese and English datasets demonstrate that our proposed method significantly enhances the zero-shot performance of existing LLMs in NER, EE, and RE tasks.

The paper is organized as follows: Sect 2 reviews and discusses related work. Sect 3 presents the design details of the ChunkUIE model proposed in this paper. Sect 4 describes the experimental program, including the dataset, evaluation metrics, and implementation details, quantitative evaluation results, and analysis. Sect 5 concludes.

## 2 Related works

### 2.1 Information extraction models

Large language models have demonstrated significant performance improvements across various language tasks, with some models capable of supporting multiple tasks simultaneously. In the domain of information extraction, these models also hold promise for addressing the generalization challenges associated with unseen labels. Lu’s study [9] seeks to adaptively adjust the structure and requirements of information extraction by incorporating schemas into instructions, resulting in a versatile model that has become a mainstream approach in subsequent UIE research.

Fine-tuning open-source LLMs is currently a prevalent method in the information extraction field. For instance, InstructUIE [10] employs the 11B FlanT5 [14] as its backbone, achieving performance comparable to ChatGPT-3.5 [15] through instruction-based fine-tuning. Similarly, YAYI-UIE [16] utilizes the Baichuan2-13B [17] backbone model, enhancing performance on NER, RE, and EE tasks through dialog-augmented instruction fine-tuning. Research by USM [18] validates that smaller LLMs can also perform well on information extraction tasks, indicating that the interplay between task complexity and model size warrants further investigation.

Several studies have also explored improving model performance through prompt learning or synthetic data generation. However, the aforementioned research has largely overlooked the potential discrepancies between the instructions used during training and those encountered in practical applications, which may impair model performance. To tackle this issue, we propose a random chunk schema construction method and validate its effectiveness through experimental results.

### 2.2 Information extraction datasets

Large-scale pre-trained corpora are essential for the effectiveness of LLMs, as they provide a wealth of knowledge and serve as a foundation for language comprehension. Although annotated datasets for information extraction tasks are relatively abundant, there is considerable variation in their labeling patterns. Several studies have addressed this issue by focusing on aspects such as size, distribution, and schema standardization [19–22], including initiatives like InstructIE [22], IEPILE [23], KnowCoder [24], etc.

In accordance with existing research practices, we sampled datasets from the domains of named entity NER, RE, and EE in both Chinese and English. Then clean the sampled data to remove duplicates and low-quality samples. The cleaned datasets were subsequently mixed, and a chunk-based schema dataset construction strategy was employed to build the training set. This training set was utilized to finetune the Qwen2-7B-Chat [11] and Llama3.1-8B-Chat [12] models. For evaluation, we used a comprehensive test set consistent with existing research to ensure a fair assessment.

## 3 Method

**Fig 1:**
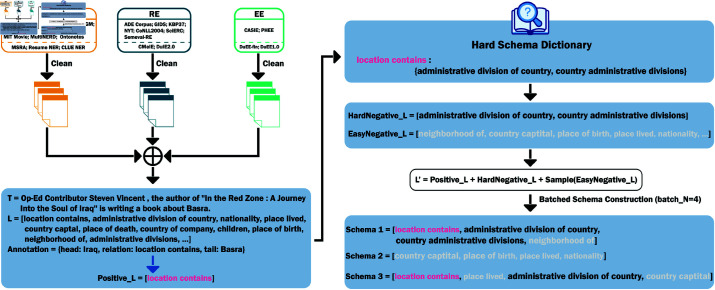
The schematic diagram of the proposed ChunkUIE, including data cleaning and chunked schema construction.

Fig 1 illustrates a schematic representation of the method proposed in this paper, using the NER task as an example. The process begins with the collection and cleaning of datasets for the NER, RE, and EE tasks, followed by the integration of the cleaned datasets. The annotations present in the text are treated as positive labels, while the predefined hard schema dictionary is used to derive hard and easy negative labels. Hard negative labels refer to labels that are semantically similar to positive labels or that have similar compositions of label words. Hard and easy judgments are defined by humans. For example, in Fig 1, the labels “administrative division of country” and “country administrative divisions” may pose challenges for determining location, and are therefore classified as difficult. We propose a carefully designed label mixing strategy along with a chunk-splitting approach to construct the final schema. This method significantly enhances the quality of the processed data and addresses the inconsistency in the number of schemas between the training and evaluation phases. The following contents will provide a detailed overview of the data processing methods and the generation of chunked instructions.

To uniformly model the IE tasks, including NER, RE and EE, we formalize these tasks by the following Eq (1):

O=ChunkUIE(Instruction,I), (1)

where Instruction comprises a natural language text sequence that includes three key elements: task type, task option, and output format. It provides a description of the task type to clearly specify the task, a description of the task option to delineate the range of labels for the output, and a description of the desired output format. The input *I* consists of a textual instance of the information extraction tasks, which is presented to the large language model alongside the instruction. The model then generates the output based on the constraints outlined in the instruction. The output *O* is a sentence that represents the structured information extracted from the input text. ChunkUIE framework employs JSON as the output format for all IE tasks.

### 3.1 Data composition and cleaning

**Fig 2:**
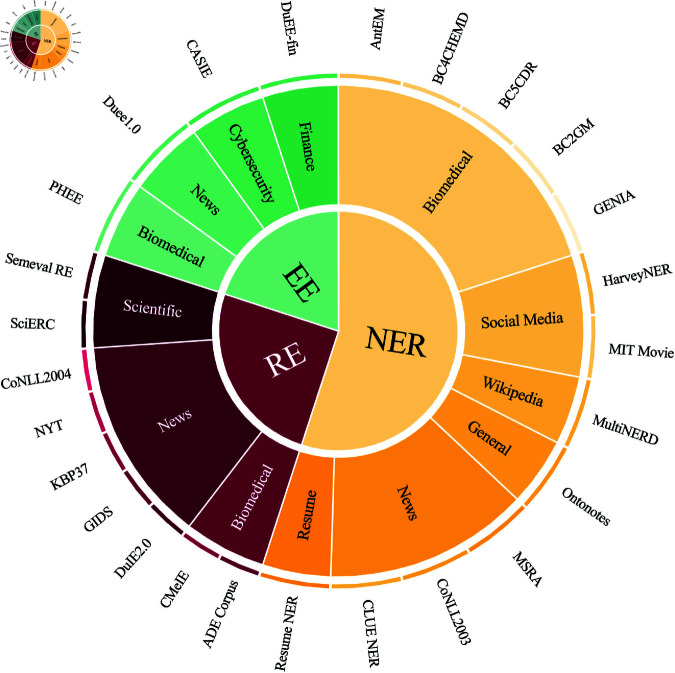
Distribution of different tasks, domains, and source datasets.

**Data Collection.** To meet the demands of various fields and practical applications, this paper focuses on the tasks of NER, RE, and EE within information extraction. We have collected datasets from multiple sources, resulting in a curated collection that includes both Chinese and English corpora. We express our gratitude for existing high-quality research in information extraction datasets, such as UIE [9], IEINSTRUCTIONS [25], and YAYI-UIE [16]. The dataset presented in this paper is meticulously crafted based on our proposed strategy, further refining the collected datasets. Previous research has demonstrated that the quality of the dataset significantly impacts the performance of supervised fine-tuning [22]. The dataset is shown in Fig 2.

Specifically, the NER task includes 13 datasets, comprising 10 English datasets. The 10 English datasets are CoNLL2003 [20], Ontonotes [26], MultiNERD [27], MIT Movie [28], HarveyNER [29], GENIA [30], BC2GM [31], BC5CDR [32], BC4CHEMD [31], and AnatEM [33]. Additionally, 3 Chinese datasets are MSRA [34], Resume NER [35], and CLUE NER [36]. The RE task encompasses 9 datasets, of which 7 are English: SemevalRE [37], SciERC [21], CoNLL2004 [38], NYT [39], KBP37 [40], GIDS [41], and ADE Corpus [42]. The two Chinese datasets for this task are DUIE2.0 [37] and CMeIE [43]. For the EE task, there are 4 datasets. Two English datasets are PHEE [44] and CASIE [45]. The other two Chinese datasets are DuEE1.0 [46] and DuEE-fin [47]. These datasets cover multiple domains, including general, medical, financial, social media, news, resume, and scientific fields.

**Data Cleaning.** First, to address the issue of duplicate samples, we implemented a deduplication process based on the text’s duplication rate. The deduplication rules are as follows:

1. Drop text with more than 70% repeated characters.
2. Drop texts shorter than ten characters without any labels.
3. Drop texts containing a high prevalence of stopwords (*e.g.*, “the”, “of”) exceeding 80%.

After deduplication, we found that some samples in the processed dataset still exhibited poor quality. To mitigate this, we manually filtered out samples that were deemed too low quality. Inspired by InstructUIE, we aimed to map the labels from different datasets into a unified schema as much as possible. This process contributes to the further enhancement of data quality, which we believe will be beneficial for model training and fine-tuning. Inspired by the research of Zhiqiang Hu *et al*., we utilize a subset of 132K samples to fine-tune the 7 billion parameter LLM. The implementation involves independent stratified sampling, where each minimum unit of the dataset is defined as a layer, denoted as $Dataset_{NER}^{i}$. So the subset of NER task can be defined as Eq (2).

$$Dataset_{NER}={⋃}_{i=1}^{k}0.2\times |Dataset_{NER}^{i}|,$$ (2)

where k is the number of NER datasets, and $Dataset_{NER}^{i}$ is the dataset one of NER datasets, such as CoNLL2003 and Ontonotes. 0.2 × $\left|Dataset_{NER}^{i}\right|$ represents randomly selecting 20% from the dataset without duplication. Similarly, define the EE and RE subsets in a similar way:

$$Dataset_{EE}={⋃}_{i=1}^{k}0.2\times |Dataset_{EE}^{i}|;$$ (3)

$$Dataset_{RE}={⋃}_{i=1}^{k}0.2\times |Dataset_{RE}^{i}|.$$ (4)

Then the final dataset is constructed by combining the NER, EE, and RE datasets, as shown in Eq (5).

$$Dataset_{F}=Dataset_{NER}\cup Dataset_{EE}\cup Dataset_{RE}.$$ (5)

Independent stratified sampling enhances the representativeness of samples by ensuring that each layer accurately reflects the characteristics of its corresponding population segment. Furthermore, this method guarantees that the number of samples in each layer is proportional to the population, thereby minimizing sampling errors.

### 3.2 Chunked instruction generation

Instructions are crucial for IE task, and three key components are essential within following instructions. (1) Task Description. This specifies the particular category of the IE task. (2) Source Text. This is the text from which the information is to be extracted. (3) Schema. This outlines the specific information to be extracted, such as entities, relationships, and events. Among these three components, the schema is particularly critical due to its flexibility and its role in guiding the model towards the specific information required for the task. Consequently, constructing an appropriate schema is vital for enhancing the robustness of model performance.

The following sections will provide a detailed overview of the processes for constructing positive and negative schemas, as well as the methodology for creating chunked instructions.

#### 3.2.1 Postive and negative schema construction.

The UIE introduces the concept of integrating a schema sequence, referred to as the structural schema instructor (SSI). Many studies have followed the SSI approach. However, this method typically utilizes all predefined labels from the dataset as SSI during the training phase. This results in a large number of labels being included in the training process, which can weaken the model’s ability to distinguish between similar but semantically different labels when faced with complex samples. Additionally, inconsistencies in the number and distribution of schemas specified during evaluation compared to training can severely harm model performance. For example, using 20 schemas during training but only 5 or 10 during evaluation creates a mismatch.

To tackle this issues, fine-tuning the number and composition of labels in the introduction during training is valuable. In this study, the schema sequence continues to follow the SSI concept. However, unlike existing research that commonly employs all labels to construct instructions, we generate stable instructions with a balanced number of positive and negative labels through the use of hard negative labels and chunked schemas. We define positive and negative labels as illustrated in Fig 1. The set of all predefined labels for a dataset comprises the collection *L*. For instance, the schema “location contains” present in the annotations serves as a positive schema, while all other schemas from the predefined label set *L* are classified as negative schemas. Inspired by contrastive learning, for a given text *T*, the schemas present in its annotations form the positive schema set Positive_L, while the remaining schemas constitute the negative label set Negative_L.

To further enhance the model’s ability to distinguish between easily confused labels, we construct a hard schema dictionary *D*, which distinguishes negative labels into HardNegative_L and EasyNegative_L. Hard negative labels refer to labels that are semantically similar to positive labels or that have similar compositions of label words. HardNegative_L and EasyNegative_L are described by Eq (6) and (7), respectively.

HardNegative_L=D[Positive_L]; (6)

EasyNegative_L=L−Positive_L−HardNegative_L. (7)

Thus, the final Negative_L consists of the entire HardNegative_L and a subset of EasyNegative_L. The benefit of this approach is that it maintains the model’s ability to differentiate between easily confused labels while reducing the number of highly similar samples, ultimately improving training efficiency. The examples of hard negative are as follows:



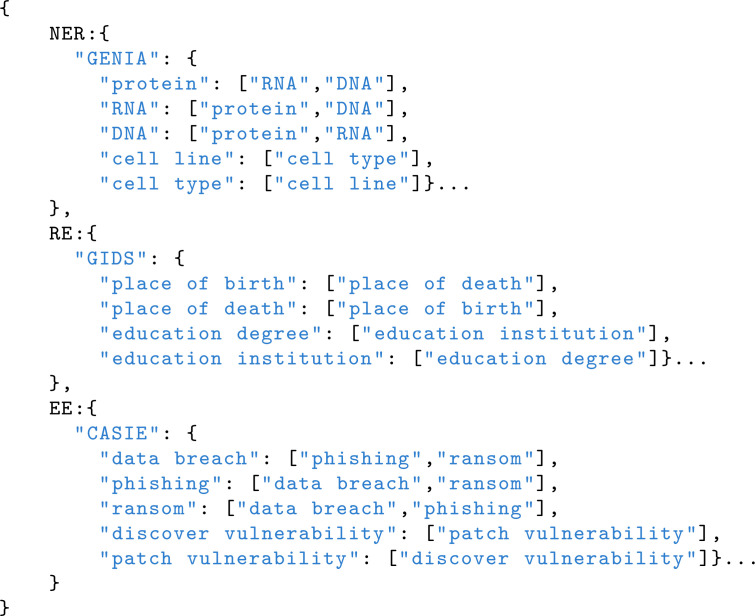



#### 3.2.2 Chunked instruction construction.

We introduce a chunked instruction construction method aimed at aligning the number of schemas during training and inference as closely as possible. Specifically, this is achieved by utilizing a dynamically adjustable parameter, split_num, to construct multiple chunked instructions from the set *L*. Consequently, the set $L^{\prime }$ will be divided into *N* chunks for querying, with each chunk querying no more than split_num schemas. The total number of resulting instructions can be described by the following Eq (8):

$$N=\left\lceil \frac{|L^{\prime }|+\text{SAMPLE}\left(L,split\_num\right)}{split\_num}\right\rceil ,$$ (8)

where *N* is the number of chunks, ⌈·⌉ is the ceiling symbol and split_num is a integer. $L^{\prime }/split\_num$ represents the division of the all labels into non-overlapping chunks. SAMPLE denotes the random sampling of split_num labels from the original set *L* to form a chunk, ensuring that each label has an equal probability of selection. This approach mitigates the issue of prioritizing the grouping of Positive_L and HardNegative_L, which could lead to performance degradation when both appear during the evaluation of the model.

The split_num is a tuning parameter that should be chosen from the range $\left[1,|L^{\prime }|\right]$. However, setting it to 1 would result in an excessively large number of training samples, adversely affecting model performance. To enhance the efficiency of both training and usage, split_num can be set as a common divisor of the schema counts across multiple datasets. In this study, split_num is set to 4. To further improve the model’s robustness, if the number of schemas in the last chunk is less than half of split_num, it is merged with the previous chunk. Otherwise, it is retained as an independent chunk.

The Sects 3.2.1 and 3.2.2 can be summarized as Algorithm 1:

**Algorithm 1 Chunked instruction generation.**



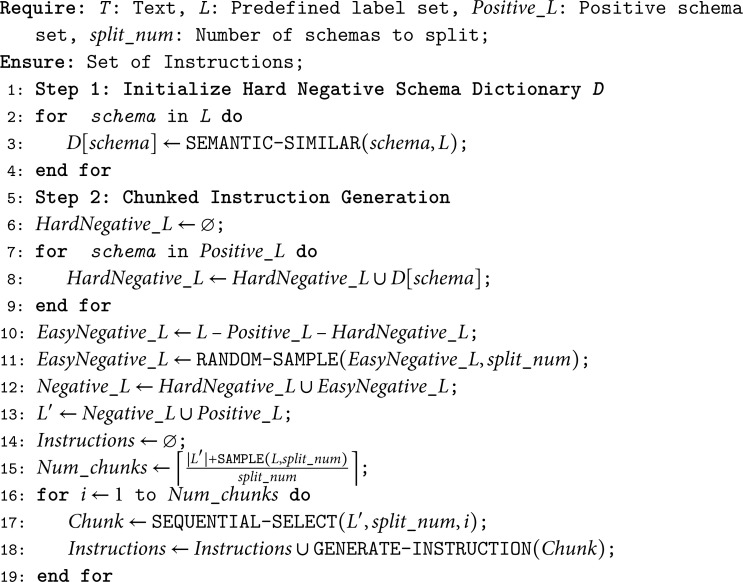



**Fig 3:**
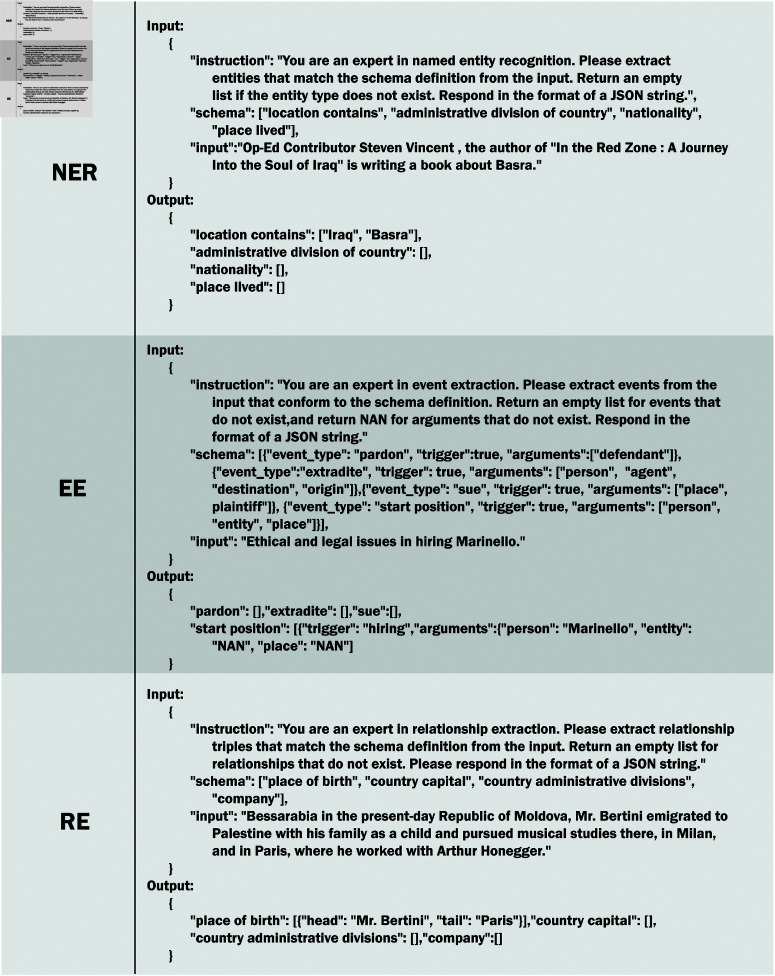
Inputs and outputs for 3 tasks: Named Entity Recognition, Event Extraction and Relation Extraction. The input and output formats adopt a structure similar to JSON strings.

For instance, if a dataset has a total of 48 schemas and a given split_num of 4, traditional methods would generate 12 unique, non-repeating instructions. However, by employing the subsets of positive labels, hard negatives, and easy negatives, this requirement can be significantly reduced to just 4 instructions. The instruction format used in chunkUIE resembles JSON strings, essentially forming a dictionary-style structure. This format comprises three main components. (1) “instruction”, which provides a task description outlining the objective of the instruction. (2) “schema”, a list of labels that need to be extracted. (3) “source text”, which is the text from which the information is to be extracted. Examples of instructions corresponding to various tasks are provided as follows Fig 3.

After constructing the chunked instructions, supervised fine-tuning technology is used to train the existing chatLLM for the IE task, which can be described by the Eq (9):

$${\text{LLM}}_{ie}=SFT({\text{LLM}}_{chat},T),$$ (9)

where ${\text{LLM}}_{ie}$ is the fine-tuned universal information extraction model. ${\text{LLM}}_{chat}$ is the fin-tuned chat model, such as the qwen2-7B model. *T* is the information extraction corpus.

## 4 Experiments

In this section, we provide a detailed illustration of the datasets, evaluation metrics, models, experimental parameters, and results utilized in this study. Through comparative and ablation experiments, we demonstrate that the proposed method enhances the zero-shot performance of LLMs on NER, RE, and EE tasks across multiple datasets.

### 4.1 Datasets and evaluation metric

**Training Datasets.** For the NER task, we utilize 13 datasets, which include 10 English datasets: CoNLL2003 [20], Ontonotes [26], MultiNERD [27], MIT Movie [28], HarveyNER [29], GENIA [30], BC2GM [31], BC5CDR [32], BC4CHEMD [31], and AnatEM [33]. Additionally, there are 3 Chinese datasets: MSRA [34], Resume NER [35], and CLUE NER [36]. The RE task encompasses 9 datasets. Seven English datasets are SemevalRE [37], SciERC [21], CoNLL2004 [38], NYT [39], KBP37 [40], GIDS [41], and ADE Corpus [42], Two Chinese datasets are DUIE2.0 [37] and CMeIE [43]. For the EE task, there are 4 datasets. Two English datasets are PHEE [44] and CASIE [45]. Two Chinese datasets are DuEE1.0 [46] and DuEE-fin [47]. These datasets cover a wide range of domains, including general, medical, financial, social media, news, resumes, and scientific fields. According to the method outlined in Sect 3, the datasets are processed and mixed, resulting in a total of 132715 sentences. During the training process, 26543 of them are used as test sets, and the rest are used as training sets. The task distribution, domain, schemas,language, and sample number of the train dataset are shown in Table 1.

**Table 1:** Statistical data of train datasets.

Task	Dataset	Domain	#Schemas	#Sentences	Language
NER	AnatEM	Biomedical	1	1133	English
	BC4CHEMD	Biomedical	1	6097	English
	BC5CDR	Biomedical	2	909	English
	BC2GM	Biomedical	1	2478	English
	GENIA	Biomedical	5	2993	English
	HarveyNER	Social Media	4	710	English
	MIT Movie	Social Media	12	1941	English
	MultiNERD	Wikipedia	16	26124	English
	Ontonotes	General	18	10998	English
	CoNLL2003	News	4	2522	English
	MSRA	News	3	8100	Chinese
	CLUE NER	News	10	1934	Chinese
	Resume NER	Resume	8	759	Chinese
EE	CASIE	Cybersecurity	5	746	English
	PHEE	Biomedical	2	579	English
	DuEE-fin	Finance	13	1403	Chinese
	Duee1.0	News	65	1403	Chinese
RE	ADE Corpus	Biomedical	1	683	English
	CMeIE	Biomedical	53	2867	Chinese
	GIDS	News	4	1705	English
	KBP37	News	18	3182	English
	NYT	News	24	10882	English
	CoNLL2004	News	5	184	English
	DuIE2.0	News	49	34225	Chinese
	SciERC	Scientific	7	273	English
	Semeval RE	Scientific	10	1295	English

**Testing Datasets.** NER task utilizes CrossNER [48], Boson (https://github.com/InsaneLife/), and Weibo [49] datasets. The experimental results of the CrossNER dataset are the average of the five subsets. EE task utilizes WikiEvents [50], RAMS [51], CrudeOil [52], FewFC [53], and CCFLaw (https://aistudio.baidu.com/projectdetail/4201483) datasets. RE task utilizes FewRel [54], SKE2020 (https://aistudio.baidu.com/datasetdetail/177191), COAE2016 (https://github.com/Sewens/COAE2016), and IPRE [55] datasets. The task distribution, domain, schemas,language, and sample number of the test dataset are shown in Table 2.

**Table 2:** Statistical data of test datasets.

Task	Dataset	Domain	#Schemas	#Sentences	Language
NER	CrossNER_Politics	Politics	9	650	English
	CrossNER_Literature	Literary	12	416	English
	CrossNER_Music	Musical	13	465	English
	CrossNER_AI	AI	14	431	English
	CrossNER_Science	Scientific	17	543	English
	Boson	News	6	191	Chinese
	Weibo	News	4	258	Chinese
EE	WikiEvents	Wikipedia	31	249	English
	RAMS	News	106	887	English
	CrudeOil	News	18	356	English
	FewFC	Finance	5	2879	Chinese
	CCFLaw	Law	9	971	Chinese
RE	FewRel	Wikipedia	100	17291	English
	SKE2020	News	49	3601	Chinese
	COAE2016	General	9	971	Chinese
	IPRE	General	35	3340	Chinese

**Evaluation Metrics.** We utilize span-based Micro-F1 as the primary metric for evaluating model performance. The Micro-F1 can be defined as Eq (12). For the NER task, the model must accurately identify both the boundaries of entities and their corresponding types. In the RE task, it is essential for the model to precisely determine the subject and object entities within a relation, along with the type of relation between them. For the EE task, we independently match event triggers, referred to as Trigger, and their associated arguments, denoted as Argument.

$$micro\text{-}Precision=\frac{\sum_{i=1}^{N}TP_{i}}{\sum_{i=1}^{N}TP_{i}+\sum_{i=1}^{N}FP_{i}};$$ (10)

$$micro\text{-}Recall=\frac{\sum_{i=1}^{N}TP_{i}}{\sum_{i=1}^{N}TP_{i}+\sum_{i=1}^{N}FN_{i}};$$ (11)

$$micro\text{-}F1=2\times \frac{micro\text{-}Precision\times micro\text{-}Recall}{micro\text{-}Precision+micro\text{-}Recall}.$$ (12)

*TP*_*i*_ is the true positives in the i-th samples. *FP*_*i*_ is the false positives in the i-th samples. *FN*_*i*_ is the false negatives in the i-th samples.

### 4.2 Models and settings

To evaluate the zero-shot generalization capabilities, we selected several prominent models for comparative analysis:

1. UIE [9]: A unified text-to-structure generation framework capable of modeling various IE tasks generically.
2. LLaMA2 [56]: A series of LLMs ranging from 7 billion to 70 billion parameters.
3. Baichuan2 [17]: A collection of multilingual LLMs available in 7 billion and 13 billion parameter configurations.
4. Qwen1.5 [57]: A comprehensive series of language models that includes distinct models with varying parameter counts.
5. Qwen2 [11]: A newer comprehensive series of language models that includes distinct models with varying parameter counts compared to Qwen1.5.
6. Mistral [58]: A 7-billion-parameter LLM designed for efficient performance.
7. ChatGPT [15]: Also known as GPT-3.5-turbo, this model represents the most advanced artificial intelligence language model to date, optimized for conversational applications.
8. LLaMA3.1 [12]: The latest release in the LLaMA model series, achieving significant improvements across various benchmarks.
9. InstructUIE [10]: A unified IE framework based on multi-task instruction tuning.
10. YAYI-UIE [16]: An end-to-end universal information extraction framework that supports both Chinese and English.

We employ the LoRA [13] technique to accomplish instruction tuning. In LoRA fine-tuning, the low-rank adjustment of the original weight matrix *W* is accomplished by training two smaller matrices, *A* and *B*, within the target module. LoRA enhances the model by incorporating two sequential low-rank matrices to approximate the residual weights. The forward computation of the adapted module is expressed as Eq (13).

$$\hat{y}=y+\Delta y=Wx+BAx.\;\text{Here,}\;A\in R^{d\times r},B\in R^{r\times k},r\ll min(d,k),$$ (13)

where $A\in R^{d\times r}$, $B\in R^{r\times k}$, and r≪min(d,k). The rank *r* of LoRA is typically much smaller than the dimensions of the original model, allowing for rapid fine-tuning with only a minimal increase in model weights, usually between 0.1% and 1%. This approach ensures low storage and memory requirements. LoRA is broadly applicable, particularly in dense projection layers of transformer architectures. By employing this technique, LoRA enhances the efficiency and cost-effectiveness of the fine-tuning process while preserving the performance of the original model. This method is especially advantageous for large language models that require frequent updates or adjustments for specific tasks.

The experiments were conducted on a platform equipped with 2 Intel Xeon E5-2683 CPU and 4 NVIDIA GeForce RTX 2080Ti GPUs. Detailed configurations of the hyperparameters during the fine-tuning process are summarized in Table 3.

**Table 3:** Training Hyperparameters.

Hyperparameter	Value
Epochs	4
Learning Rate	5.00E-05
Batch Size	2
Accumulate	4
Optimizer	AdamW
Lora_r	16
Lora_alpha	32
Lora_dropout	0.05

### 4.3 Main experimental results and analysis

Tables 4 and 5 present the F1 zero-shot performance across three tasks and two languages. Notably, the tested models include those with 7 billion and 13 billion parameters, as well as closed-source models like ChatGPT. Overall, our proposed method demonstrates a significant performance improvement for the 7 billion parameter open-source models after supervised fine-tuning, achieving performance that is comparable to, or even better than, ChatGPT.

**Table 4:** Zero-shot performance on English datasets. UIE necessitates predefined entity types; given that such information is not provided by the FewRel dataset, we can’t evaluate UIE’s performance on the FewRel dataset. For the task of event extraction, we only present the results of event detection in the main text. The best value in each metric is denoted in bold, and the second-best score is highlighted with an underline.

Method	NER	EE				RE
	CrossNER	WikiEvents	RAMS	CrudeOil	Avg	FewRel
LLama2-7B	1.37	0	0	0	0	0
Llama3.1-8B	13.81	0	0	0	0	0
Qwen2-7B	42.80	0	0.16	0	0.05	0
Mistral-7B	42.83	0	0	0	0	6.84
Qwen1.5-14B	50.13	0	0	0	0	7.82
ChatGPT	**58.37**	2.95	8.35	1.41	4.23	9.96
UIE	38.37	5.12	9.25	6.45	6.94	-
InstructUIE-11B	49.36	**11.64**	**24.27**	23.26	19.72	**39.55**
YAYI-UIE-13B	50.39	10.97	18.87	12.45	14.09	36.09
ChunkUIE-Llama3.1-8B	58.13	8.67	19.71	34.52	20.96	32.23
ChunkUIE-Qwen2-7B	57.20	11.45	15.69	**36.65**	**21.26**	28.50

**Table 5:** Zero-shot performance on Chinese datasets. The best value in each metric is denoted in bold, and the second-best score is highlighted with an underline.

Method	NER			EE			RE			
	Boson	Weibo	Avg	FewFC	CCF Law	Avg	SKE2020	COAE2016	IPRE	Avg
LLama2-7B	8.19	2.43	5.31	0	0	0	0	0	0	0
Llama3.1-8B	44.64	31.87	38.26	0	0	0	0	0	0	0
Qwen2-7B	38.81	35.63	37.22	0	0	0	0	0	0	0
Mistral-7B	29.13	10.02	19.58	4.69	0.23	2.46	6.84	5.24	0.82	4.30
Qwen1.5-14B	26.49	25.34	25.92	11.47	3.25	7.36	7.69	11.97	2.16	7.27
ChatGPT	38.53	29.30	33.92	16.15	0	8.08	24.47	19.31	6.73	16.84
YAYI-UIE-13B	49.25	36.46	42.86	**81.28**	12.87	47.08	70.80	19.97	22.97	37.91
ChunkUIE-Llama3.1-8B	**59.00**	35.11	47.06	79.75	**61.41**	**70.58**	70.91	48.20	28.13	49.08
ChunkUIE-Qwen2-7B	56.83	**41.20**	**49.02**	73.13	60.20	66.67	**71.46**	**51.36**	**31.27**	**51.36**

From Table 4, it is evident that for the NER task on English datasets, ChunkUIE-Llama3.1-8B ranks second in performance, showing only a small gap compared to ChatGPT. Both ChunkUIE-Llama3.1-8B and ChunkUIE-Qwen2-7B significantly outperform most other models in English NER tasks. This confirms the effectiveness of our proposed method and highlights the value of incorporating a larger proportion of NER training data in the training dataset. In the EE task, ChunkUIE-Llama3.1-8B and ChunkUIE-Qwen2-7B demonstrate significantly better performance than models that were not fine-tuned on IE tasks, which can be attributed to the effectiveness of supervised fine-tuning. Although the performance on the WikiEvents and RAMS datasets slightly lags behind that of InstructUIE, this may be due to the advantages of using larger models. Nonetheless, the average performance in the EE task still favors ChunkUIE-Llama3.1-8B and ChunkUIE-Qwen2-7B. For the RE task, both ChunkUIE-Llama3.1-8B and ChunkUIE-Qwen2-7B outperform the models that were not fine-tuned, demonstrating the efficacy of fine-tuning. However, their performance is inferior to that of InstructUIE and YAYI-UIE, which may be influenced by the benefits of larger models.

From Table 5, it is evident that for the NER task on Chinese datasets, ChunkUIE-Qwen2-7B achieves the best average performance, while ChunkUIE-Llama3.1-8B underperforms. This discrepancy may be attributed to differences in the distribution of the fine-tuning data used for the Chinese NER task in this study compared to the data on which the Qwen model was trained. In the EE task, both ChunkUIE-Llama3.1-8B and ChunkUIE-Qwen2-7B significantly outperform other models, further validating the effectiveness of our proposed method. For the RE task, both models also lead in performance compared to others, demonstrating the efficacy of our approach and the effectiveness of supervised fine-tuning.

Overall, ChunkUIE-Qwen2-7B and ChunkUIE-Llama3.1-8B exhibit strengths and weaknesses across the three tasks in both Chinese and English. However, ChunkUIE-Qwen2-7B shows a distinct advantage in Chinese tasks, aligning with the inherent strengths of the Qwen2 model in handling Chinese language data. Through the above quantitative evaluations, we confirm that our method enhances SFT performance on both LLaMA and Qwen models, resulting in models that are comparable to, or even outperform, ChatGPT in information extraction tasks. This further validates the effectiveness of our proposed approach.

### 4.4 Ablation study

To validate the impact of hard negative samples, we conducted ablation experiments. Specifically, we constructed Negative_L without using the negative dictionary, instead treating all labels apart from Positive_L as EasyNegative_L. The remainder of the process remained unchanged. Then rebuilt the training set and fine-tuned the Qwen2-7B and LLaMA3.1-8B models using the LoRA technique. A fair comparison was achieved by employing the same testing datasets. The experimental results are presented in Tables 6 and 7. The same metric is used to quantitatively validate our method’s effectiveness.

**Table 6:** Ablation experiment of removing hard negative schema dictionary on English datasets. The best value in each metric is denoted in bold.

Method	NER	EE				RE
	CrossNER	WikiEvents	RAMS	CrudeOil	Avg	FewRel
ChunkUIE-Llama3.1-8B	**58.13**	8.67	19.71	34.52	20.96	**32.23**
w/o Hard	55.92	6.73	**20.11**	30.87	19.24	29.25
ChunkUIE-Qwen2-7B	57.20	11.45	15.69	**36.65**	**21.26**	28.50
w/o Hard	57.00	**14.33**	14.68	33.34	20.78	25.17

**Table 7:** Ablation experiment of removing hard negative schema dictionary on Chinese datasets. The best value in each metric is denoted in bold.

Method	NER			EE			RE			
	Boson	Weibo	Avg	FewFC	CCF Law	Avg	SKE2020	COAE2016	IPRE	Avg
ChunkUIE-Llama3.1-8B	**59.00**	35.11	**47.06**	**79.75**	**61.41**	**70.58**	70.91	48.20	28.13	49.08
w/o Hard	58.27	33.42	45.84	78.19	52.17	65.18	70.85	49.95	23.90	36.94
ChunkUIE-Qwen2-7B	38.81	**35.63**	37.22	73.13	60.20	66.67	**71.46**	**51.36**	**31.27**	**51.36**
w/o Hard	33.75	32.23	32.99	71.63	51.35	61.49	71.42	50.93	30.43	50.92

From Tables 6 and 7, it is evident that the use of a hard negative dictionary enhances model performance across most datasets. Specifically, both ChunkUIE-Qwen2-7B and ChunkUIE-Llama3.1-8B benefit from hard negatives in the NER task, likely due to the clear boundaries characteristic of entity recognition. In the EE task, the performance improvements for ChunkUIE-Qwen2-7B and ChunkUIE-Llama3.1-8B are limited. However, there is a more pronounced enhancement in performance for the RE task.

To validate the effectiveness of ChunkUIE in fine-tuning the baseline model for information extraction tasks, we present ablation study results on English and Chinese datasets in Tables 8 and 9, respectively. As shown in Table 8, the Llama3.1-8b model achieves significant performance improvements in NER, EE, and RE tasks after fine-tuning with ChunkUIE. While the qwen2-7B model already demonstrates strong initial NER performance, it also achieves notable gains across all tasks following the application of ChunkUIE. As shown in Table 8, it can be seen that for the Chinese dataset, the performance of the model fine-tuned with chunkUIE is also significantly improved.

**Table 8:** Ablation experiment of removing chunked instruction on English datasets.

Method	NER	EE				RE
	CrossNER	WikiEvents	RAMS	CrudeOil	Avg	FewRel
ChunkUIE-Llama3.1-8B	58.13	8.67	**19.71**	34.52	20.96	**32.23**
w/o ChunkUIE Finetune	13.81	0	0	0	0	0
ChunkUIE-Qwen2-7B	57.20	**11.45**	15.69	**36.65**	**21.26**	28.50
w/o ChunkUIE Finetune	42.80	0	0.16	0	0.05	0

**Table 9:** Ablation experiment of removing chunked instruction on Chinese datasets.

Method	NER			EE			RE			
	Boson	Weibo	Avg	FewFC	CCF Law	Avg	SKE2020	COAE2016	IPRE	Avg
ChunkUIE-Llama3.1-8B	**59.00**	35.11	47.06	**79.75**	**61.41**	**70.58**	70.91	48.20	28.13	49.08
w/o ChunkUIE Finetune	44.64	31.87	38.26	0	0	0	0	0	0	0
ChunkUIE-Qwen2-7B	56.83	**41.20**	**49.02**	73.13	60.20	66.67	**71.46**	**51.36**	**31.27**	**51.36**
w/o ChunkUIE Finetune	38.81	35.63	37.22	0	0	0	0	0	0	0

The qwen and llama used in this study use a decoder-only design, which has stronger generation capabilities than the model using the encoder. The long sequence modeling capability of the model using only the decoder is very beneficial for information extraction tasks because the information to be extracted may be distributed in different sentences in a paragraph.

Overall, the introduction of contrastive learning principles significantly enhances the model’s ability to distinguish between easily confused terms, leading to improved performance across NER, RE, and EE tasks.

## 5 Conclusion

LLMs exhibit significant advantages in information extraction tasks. In this paper, we introduce ChunkUIE, which involves cleaning existing information extraction datasets and proposing a chunk-based random schema construction method. Additionally, by constructing challenging samples, we mitigate the model’s semantic confusion regarding similar patterns. Experiments on multiple datasets using supervised fine-tuning with LLaMA3.1-8B and Qwen2-7B validate the effectiveness of our proposed method in enhancing zero-shot performance for information extraction. Due to computational resource constraints, this study primarily investigates the fine-tuning effects of LLMs with approximately 7 billion parameters, leaving the specific performance on models of other sizes unclear. Exploring the data scalability of models of different sizes by gradually increasing the amount of data is one of the directions worth studying. Future work could focus on further enhancing dataset quality while investigating the information extraction capabilities of larger LLMs.
